# Cavo-Atrial Inferior Vena Cava (IVC) Restoration With Polytetrafluoroethylene Graft: Overcoming Lethal Injuries

**DOI:** 10.7759/cureus.74099

**Published:** 2024-11-20

**Authors:** Evangelia Florou, Upali Anand, Emeema Govindu, Stephen Gregory, Andreas Prachalias

**Affiliations:** 1 Hepato-Pancreato-Biliary Surgery, King's College Hospital, London, GBR; 2 Hepato-Pancreato-Biliary Interventional Radiology, King's College Hospital, London, GBR; 3 Hepato-Pancreato-Biliary Surgery and Liver Transplantation, London Bridge Hospital, London, GBR

**Keywords:** abdominal trauma, bleeding, hepatic vein obstruction, hepatocaval confluence, inferior vena cava, noncompressive torso hemorrhage, polytetrafluoroethylene graft, suprahepatic inferior vena cava, vascular injury, venovenous bypass

## Abstract

Injuries to the inferior vena cava (IVC) carry high risks and mortality rates. We present a case of suprahepatic IVC injury that was successfully treated with polytetrafluoroethylene (PTFE) graft insertion without cardiopulmonary bypass.

A 46-year-old woman was transferred to our trauma centre after a high-speed motor vehicle collision. Computed tomography (CT) revealed a suprahepatic IVC injury with an expanding hematoma within the diaphragmatic muscle. Via a thoracoabdominal approach, total vascular occlusion allowed evacuating the hematoma under control to allow IVC injury assessment. A large defect in the suprahepatic IVC was noted with a near-complete transection at the level of the hepatocaval confluence. A PTFE graft was successfully inserted restoring continuity between the right atrium and hepatic veins. The patient recovered and remains well two years post-trauma.

Injuries to the IVC are uncommon and often fatal. Effective coordination between specialties can facilitate positive outcomes even in the most complex clinical scenarios. IVC injuries may be survivable when managed at high-volume expert centres with intensive care, anaesthesia, and surgical expertise.

## Introduction

Noncompressive torso hemorrhage (NCTH), though potentially avoidable, remains a primary cause of early trauma fatality. Major vascular injuries, which include damage to the inferior vena cava (IVC) and aorta, account for approximately 25% of these casualties [1]. Exsanguination resulting from these injuries occurs swiftly, with death occurring within a median time of two hours from hospital admission [1].

IVC injuries remain rare entities even in large trauma centres with an estimated incidence rate of 3.2-3.9% among all trauma patients [1,2,3]. IVC injuries represent 30-40% of vascular injuries and have an overall mortality rate of 38-80% reported in the literature [2,4,5].

## Case presentation

A 46-year-old lady was involved in a high-speed motor vehicle accident. She appeared to be an unrestrained car driver struck by a lorry. She was intubated on the scene as she was found to be hypotensive and unstable. Transfusion achieved hemodynamic stability allowing transfer to our tertiary trauma centre.

A full-body computed tomography (CT) showed right hemopneumothorax, pelvic fractures (right acetabular and right iliac crest), bilateral comminuted femoral fractures, and bilateral extensive rib fractures. Liver contusion in segment 7 was assessed as grade 1, while no other injury to abdominal organs was observed.

**Figure 1:**
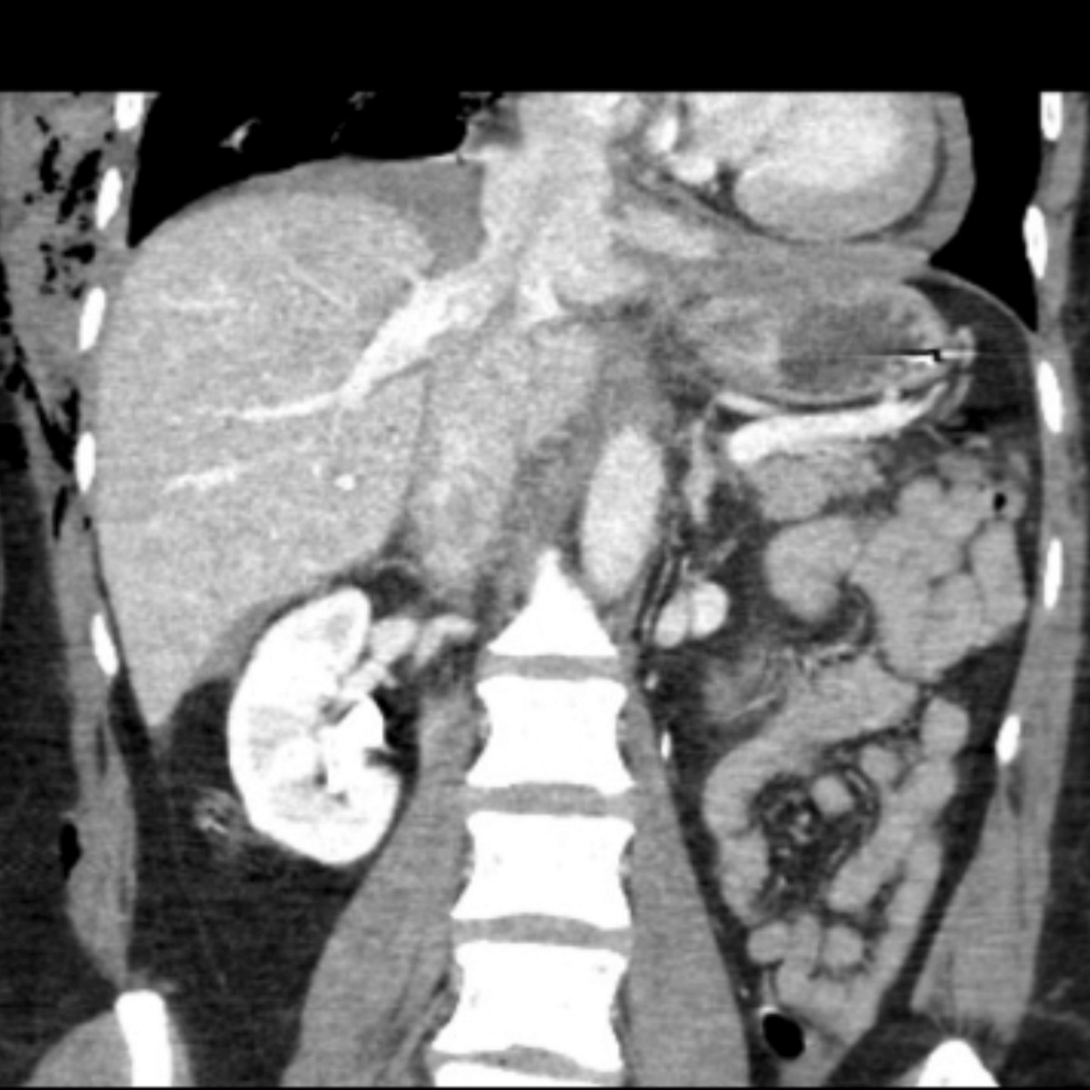
Radiological findings Coronal view of CT demonstrating IVC injury with active contrast extravasation and expanding hematoma within the diaphragmatic muscle CT: computed tomography; IVC: inferior vena cava

**Figure 2:**
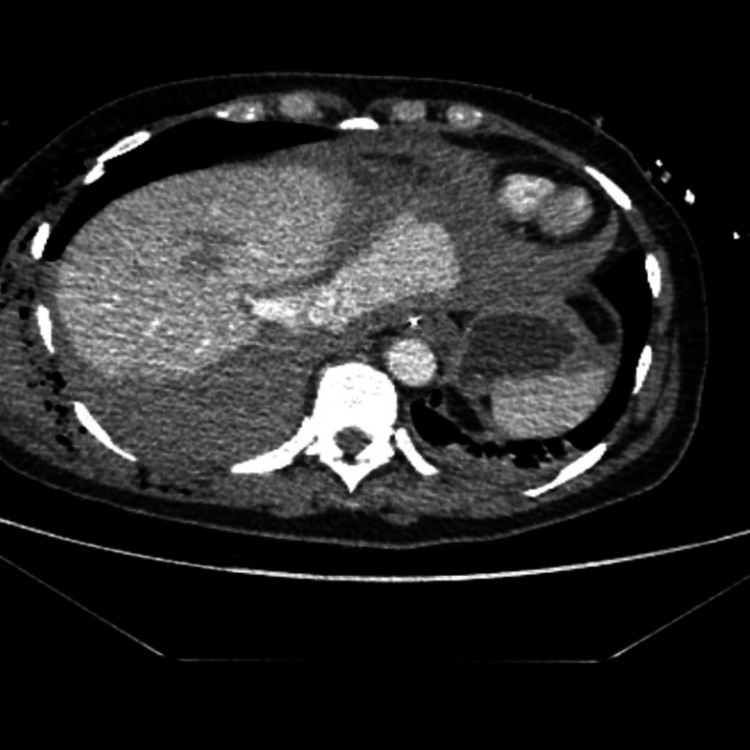
Radiological findings Axial images of CT demonstrating the active bleeding from the suprahepatic IVC injury CT: computed tomography; IVC: inferior vena cava

After successful resuscitation and in the absence of major abdominal injury, she was transferred to the intensive care unit (ICU) requiring inotropic support and renal replacement therapy. After eight hours of admission, acute hemodynamic deterioration along with hemoglobin drop led to repeat imaging. The repeat CT scan revealed a laceration at the suprahepatic portion of the IVC extending from the confluence of the hepatic veins to the right atrium. The IVC rupture was associated with surrounding hematoma but also active hemorrhage with contrast extravasation within the diaphragmatic muscle. Associated mild hemopericardium was also noted, but there were no clinical features of tamponade (Figure 1 and Figure 2).

Substantial volumes of blood transfusion and blood products were necessitated to maintain hemodynamic stability while the patient continued receiving inotropic support.

Surgery

A decision was made for immediate exploration. Cardiothoracic and hepatobiliary teams attended, and a thoracoabdominal approach was planned. After opening the chest, hemopericardium was assessed as mild. Isolation of the supradiaphragmatic, extracardiac portion of IVC was performed. On laparotomy, the abdominal cavity was free of blood, and there was no abdominal organ injury noted. The suprahepatic IVC hematoma was mainly expanding within the falciform and triangular ligaments as well as within the diaphragmatic muscle. The liver was congested as the hematoma caused compression of the hepatic veins and outflow obstruction.

The hepatoduodenal ligament was encircled in preparation to perform inflow occlusion and the Pringle manoeuvre and the infrahepatic IVC was also encircled ready to be clamped. Evacuation of the hematoma and total vascular exclusion (TVE) were performed. The evacuation of the hematoma in conjunction with TVE led to cardiac arrest. Cardiac massage and aggressive anaesthetic resuscitation measures resulted in a return of spontaneous circulation after one minute.

**Figure 3:**
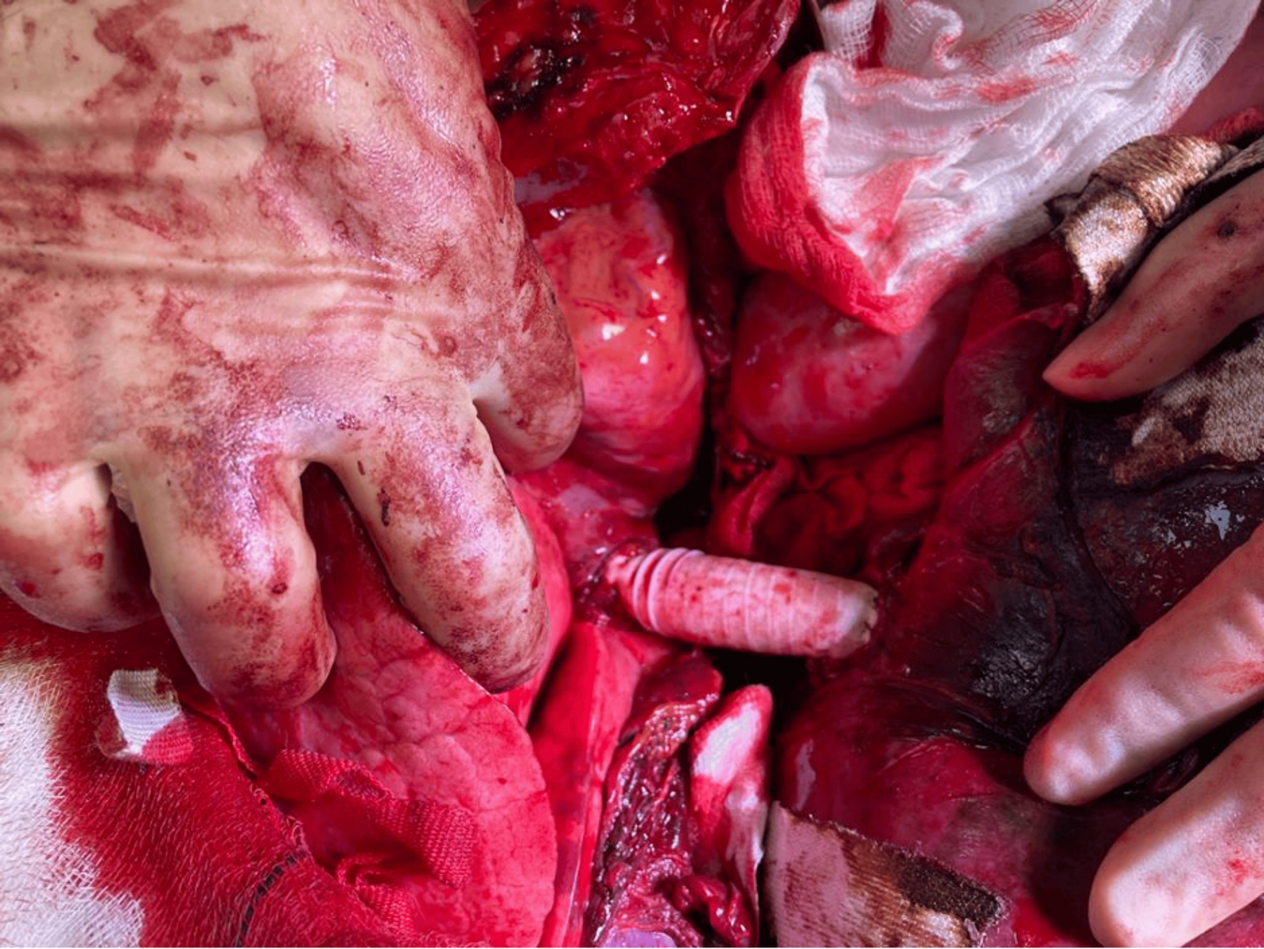
Restoration of IVC with PTFE graft The PTFE graft anastomosed to the hepatocaval confluence proximally and the right atrium distally restoring IVC continuity. The heart and lung are fully exposed, and the diaphragmatic muscle is divided to ensure adequate access IVC: inferior vena cava; PTFE: polytetrafluoroethylene

After the evacuation of the hematoma, assessment of the injury revealed an approximate 300-320-degree loss of the circumference of IVC between the right atrium and the hepatic venous confluence. A 19 mm polytetrafluoroethylene (PTFE) graft was used and anastomosed to the hepatic venous confluence followed by a proximal anastomosis to the right atrium. The TVE was reversed, and both systemic and portal circulation were restored (Figure 3).

The duration of TVE was approximately 30 minutes. A splenic rupture as a result of TVE was dealt with splenectomy. The pericardium was repaired using bovine mesh material. Cholecystectomy completed the surgical management of the patient.

At the end of the operation, the patient was hemodynamically stable and metabolically recovered which allowed for the subsequent management of the skeletal injuries.

Postoperative course

During the postoperative course, both bleeding and thrombotic complications occurred. Anticoagulation with unfractionated heparin infusion was commenced to avoid thrombotic events to the graft. Fifteen days later, severe thrombocytopenia of unclear cause led to the temporary discontinuation of unfractionated heparin and mandated platelet transfusion. Heparin-induced thrombocytopenia (HIT) screen was negative, and anticoagulation was resumed once the platelet count recovered. An episode of bleeding per rectum followed, due to an actively bleeding anal fissure, and was successfully treated endoscopically. Interval imaging revealed newly developed bilateral pulmonary embolisms attributed to deep vein thrombosis, following repeat orthopaedic procedures. There were no adverse events of thrombotic episodes related to the graft as this was assessed with imaging and Doppler studies throughout admission.

**Figure 4:**
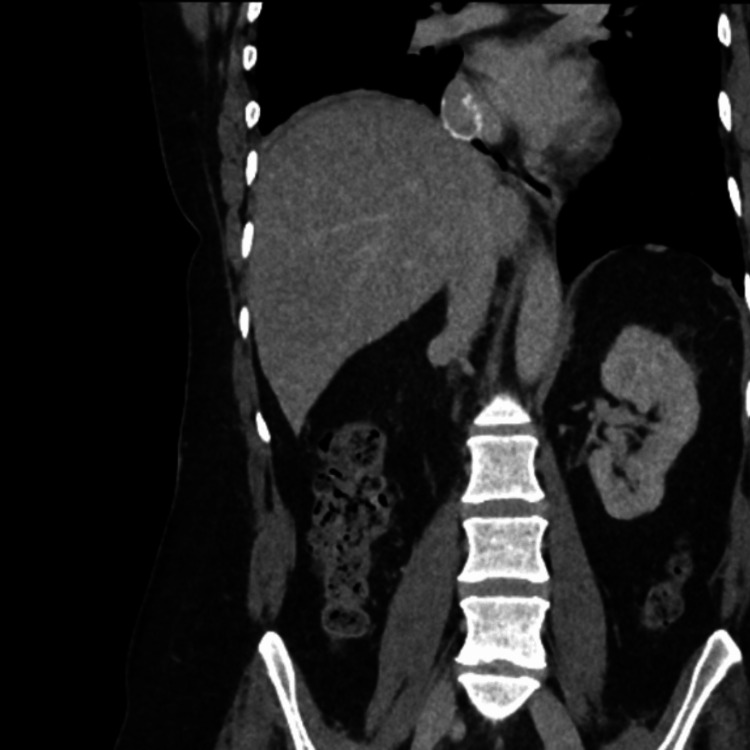
Imaging two years post-surgery: IVC restoration with PTFE PTFE-atrium patency IVC: inferior vena cava; PTFE: polytetrafluoroethylene

**Figure 5:**
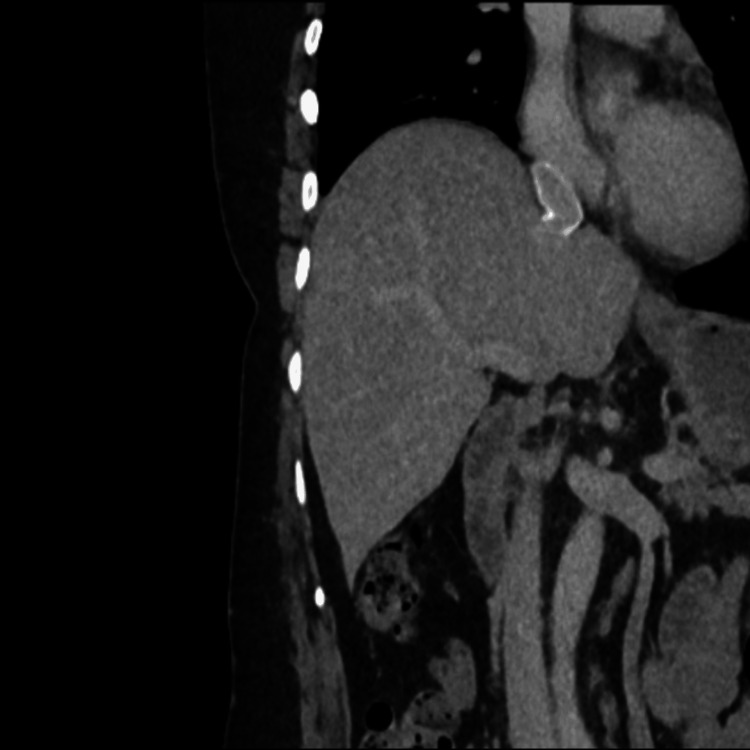
Imaging two years post-surgery: IVC restoration with PTFE PTFE-hepatic confluence patency IVC: inferior vena cava; PTFE: polytetrafluoroethylene

The patient gradually improved over a period of six weeks. She was discharged from the hospital with arrangements for physiotherapy and rehabilitation. She remains fit and well three years post-surgery and remains on lifelong anticoagulation (Figure 4 and Figure 5).

## Discussion

IVC injuries carry a mortality of 38-70% [1-3]. Only 30-50% of these cases reach trauma centres alive, and despite all efforts for resuscitation and surgical intervention, 30% die within 24 hours from admission [4,5].

Prompt and efficient prehospital care, aggressive resuscitation avoiding hypothermia and coagulopathy, as well as prompt surgical management will result in better prognosis and lower mortality in this group of patients [4,6]. However, even in large trauma centres, mortality from IVC injuries remains as high as 75%, highlighting the difficulty in applying a treatment strategy that takes advantage of radiological, endovascular, and surgical advances over the last decade [5-7].

Studies assessing the risk factors predicting high mortality indicate that low Glasgow Coma Scale (GCS) score, hypotension, thoracotomy in the emergency department or operating room, and a large amount of transfusion of red blood cells in the first 24 hours indicative of high blood loss (>7.2 litres) are all negative predicting factors of survival [4,5].

Outcomes of injuries at the craniocaudal level of IVC are conflicting in published literature. Anatomically, a vein wall injury tends to expand as bleeding is ongoing. The level of the injury across IVC may determine whether the bleeding could be potentially tamponaded by surrounding organs and structures, potentially being a contributory factor of gaining time in diagnosis and management planning. However, in the acute setting, prediction is not feasible, and injury to the adjacent IVC organs, such as the liver or kidney's vasculature, which is a common occurrence, impedes further attempts at the accurate estimation of injuries [8,9].

Proximity of the IVC injury to the heart is considered to result in higher mortality rates when compared to retrohepatic IVC injuries by some authors [4,5], while Choi et al. reported higher mortality in retrohepatic IVC injury and the lowest noted in suprahepatic IVC injury in their series. This was attributed to the blunt nature of IVC injury and the tamponade effect on the surrounding tissues, leading to more time in planning management [2].

However, the mechanism of injury was not a negative predictor factor, as reported in other studies. Similar mortality was noted in both blunt and penetrating IVC injuries in large trauma centre series [1,5,10].

The historically high mortality rates of IVC injuries reported in the literature are indicative of the severity of the injury. Future studies focusing on long survivors and factors related to their management may identify anatomical or supportive measures that act as time gainers in the challenges posed by such lethal injuries [11,12].

In our case, the IVC injury was not recognised at the initial CT. Initial stability was followed by acute deterioration, and the IVC injury was obvious only on repeat imaging. Hemodynamic support within a hospital and intensive care environment is advantageous in a tertiary trauma centre setting, while a multidisciplinary approach is possible with the involvement of specialists from radiology, anaesthesia, hepatobiliary, and cardiothoracic teams aiding better outcomes. Moreover, in this case, the self-containing, although rapidly expanding, intradiaphragmatic hematoma gave vital time for planning which was proven crucial for the final outcome.

Cardiopulmonary bypass was not deemed necessary, a suggested practice while attempting the repair of this type of injury [10-14]. A young previously healthy individual with a pending bursting hematoma and concurrent liver outflow obstruction can potentially sustain a period of TVE. Appropriate circulatory support and surgical manoeuvres at hand, such as TVE, allowed the quick assessment of the suprahepatic IVC without losing precious time of circulatory cardiopulmonary bypass in this urgent setting. To our knowledge, this is the first case report documenting the permanent successful cavo-atrial repair of a suprahepatic IVC rupture without cardiac bypass.

PTFE offers a sustainable solution for restoring vascular continuity. Patients with PTFE grafts are traditionally seen as high risk for developing graft thrombosis, a serious postoperative complication. The foreign body and low-pressure venous system are thought to contribute to thrombosis risk. While consensus is lacking, lifelong anticoagulation using coumarins is commonly practised following PTFE implantation [15-17].

## Conclusions

IVC injuries indicate a high injury severity scale where a poor prognosis is often observed. However, beyond the difficulties of hemodynamic and circulatory support to replace rapid blood loss and ensure organ oxygenation in this group of patients, factors that can gain time and permit an endeavour for surgical intervention and vascular reconstruction should be identified, as a successful surgical intervention is often the only chance of survival for these patients.

A multidisciplinary approach involving specialists from radiology, anaesthesia, hepatobiliary, and cardiothoracic teams can aid outcomes through collaboration. IVC injuries may be survivable when managed at high-volume expert centres with intensive care, anaesthesia, and surgical expertise.
